# A comprehensive DNA barcoding reference database for Plecoptera of Switzerland

**DOI:** 10.1038/s41598-024-56930-5

**Published:** 2024-03-15

**Authors:** Laurent Vuataz, Jean-Paul Reding, Alexis Reding, Christian Roesti, Céline Stoffel, Gilles Vinçon, Jean-Luc Gattolliat

**Affiliations:** 1 Département de zoologie, Palais de Rumine, Muséum cantonal des sciences naturelles, Place Riponne 6, 1005 Lausanne, Switzerland; 2 Department of Ecology and Evolution, University of Lausanne (UNIL), 1015 Lausanne, Switzerland; 3 Corcelles, Switzerland; 4 Bern, Switzerland; 5 Grenoble, France

**Keywords:** Aquatic insects, Species delimitation, Morphology, Water monitoring, Stoneflies, Biodiversity, Entomology, Classification and taxonomy, Genetic databases, Phylogenetics

## Abstract

DNA barcoding is an essential tool in modern biodiversity sciences. Despite considerable work to barcode the tree of life, many groups, including insects, remain partially or totally unreferenced, preventing barcoding from reaching its full potential. Aquatic insects, especially the three orders Ephemeroptera, Plecoptera, and Trichoptera (EPT), are key freshwater quality indicators worldwide. Among them, Plecoptera (stoneflies), which are among the most sensitive aquatic insects to habitat modification, play a central role in river monitoring surveys. Here, we present an update of the Plecoptera reference database for (meta)barcoding in Switzerland, now covering all 118 species known from this country. Fresh specimens, mostly from rare or localized species, were collected, and 151 new CO1 barcodes were generated. These were merged with the 422 previously published sequences, resulting in a dataset of 573 barcoded specimens. Our CO1 dataset was delimited in 115 CO1 clusters based on a priori morphological identifications, of which 17% are newly reported for Switzerland, and 4% are newly reported globally. Among the 115 CO1 clusters, 85% showed complete congruence with morphology. Distance-based analysis indicated local barcoding gaps in 97% of the CO1 clusters. This study significantly improves the Swiss reference database for stoneflies, enhancing future species identification accuracy and biodiversity monitoring. Additionally, this work reveals cryptic diversity and incongruence between morphology and barcodes, both presenting valuable opportunities for future integrative taxonomic studies. Voucher specimens, DNA extractions and reference barcodes are available for future developments, including metabarcoding and environmental DNA surveys.

## Introduction

DNA barcoding and, more recently, metabarcoding (e.g., community DNA metabarcoding, environmental DNA^1^) have become an essential tool in modern biodiversity sciences, accelerating and facilitating species discovery, description, identification, inventory, and monitoring, while providing useful information on species boundaries, relationships, and dynamics, among others^2^. However, the reliability of DNA (meta)barcoding largely relies on the extent and quality of reference databases that connect genetic sequences to taxonomic names^3–7^. Establishing robust reference databases is essential for efficient downstream investigations and conservation efforts, especially in the face of global biodiversity loss.

Benthic macroinvertebrates, which include taxa sensitive to anthropogenic alterations of their habitats, are extensively used for water quality monitoring^8–10^. Among them, the Ephemeroptera, Plecoptera, and Trichoptera (EPT) orders of aquatic insects, which spend their immature stages in freshwater, are globally recognized as key bioindicators^11,12^, notably through the popular biotic indexes^13–15^. Plecoptera (stoneflies), a hemimetabolous insect order of ancient origin^16–18^, holds a special place in river bioindication, as many species are considered highly sensitive to environmental changes and habitat alteration^19–21^. In Switzerland, stoneflies play an important role in the official IBCH index, where the most sensitive category comprises exclusively stonefly families^22,23^. More than 3700 stonefly species have been described globally, with ca. 500 species occurring in Europe^17^. Despite of its relatively small size, Switzerland harbors a rich diversity of stoneflies, with 125 species and two subspecies documented (including historical records). This can be attributed to the country's favorable geographic position, varied relief, and hydrography^24^. Stoneflies being mainly orophilic and crenophilic taxa, the greatest diversity of species is recorded in the headwaters of rivers Rhône, Rhine, Reuss, Inn and Ticino, all of which originate in the Swiss Alps. Despite their wide distribution, stoneflies are experiencing alarming declines in many regions^25^, including Switzerland, where around 40% of the species are classified in the national Red List^26^.

Gattolliat et al.^24^ paved the way for a barcode reference database of stoneflies occurring in Switzerland by publishing genetic barcodes for 270 specimens belonging to 90 species, out of a total of 112 reported at that time. Meanwhile, *Protonemura jurassica*^27^ and *Dictyogenus jurassicum*^28^ were newly described; *Leuctra biellensis* was reinstated^29^; *Taeniopteryx schoenemundi* was rediscovered^30^; *Zwicknia westermanni* was newly reported^31^. In addition, Roesti^30^ mentioned two undescribed species occurring in Switzerland (*Zwicknia* cf. *rupprechti* and *Nemoura* sp.). The number of stonefly species recorded in Switzerland thus has increased to 118^30^, including six additional species extinct at the national level: *Besdolus ventralis* and *Isogenus nubecula*, last mentioned by Neeracher^32^; *Xanthoperla apicalis* and *Brachyptera monilicornis*, last mentioned by Aubert^33^; *Taeniopteryx nebulosa*, last mentioned by F. Ris in 1886^34^; *Brachyptera braueri*, mentioned as extinct by Aubert^35^. *Nemoura palliventris* is considered as a potentially occurring species in Switzerland^30^.

Here we provide an updated version of the barcode reference database established by Gattolliat et al.^24^, which now includes voucher specimens, DNA extractions and barcode sequences for each stonefly species currently documented in Switzerland, following the most recent inventory available in Roesti^30^. Specifically, we raised the total number of reference specimens to 573, adding specimens from 23 missing species on the one hand, and increasing the number of populations for many previously published species on the other hand. For this, targeted sampling strategies were employed, specifically focusing on rare species or those found at their distributional limits within Switzerland. This work will support future studies in DNA (meta)barcoding and environmental monitoring, while providing recommendations for future investigations in stonefly systematics.

## Materials and methods

### Abbreviations and depositories

JPR: Jean-Paul G. Reding; AR: Alexis Reding; CR: Christian Roesti; GV: Gilles Vinçon. The investigated specimens, which were morphologically identified by three expert taxonomists (JPR, CR, GV) according to the most recent insights, are assembled in a carefully-curated collection housed in the Department of zoology, State Museum of natural sciences (Muséum cantonal des sciences naturelles) in Lausanne, Switzerland.

All barcodes newly published in this study, comprising those previously generated by AR^36^ and newly generated ones (see next section), are deposited in GenBank (OR733730–OR734007). Comprehensive information on specimens and associated barcodes, including identifications and GBIF codes, are provided in Table S2.

### Sampling and sequencing

We first combined all barcode sequences from Gattolliat et al.^24^ with unpublished barcode sequences from AR^36^. The former sequences were obtained from the Barcode of Life Data Systems (BOLD^37^ hereafter) available at www.boldsystems.org. For this, we downloaded all the sequences of the project code PLEAA (title “Plecoptera–Trichoptera of Switzerland”) and manually deleted all sequences of trichopteran species. All sequences from species not reported from Switzerland according to Roesti^30^ were then excluded.

To complement our dataset, specimens from missing species or populations were then newly collected in the field or selected within the personal collections of JPR and CR. For these, total genomic DNA was extracted using the BioSprint 96 extraction robot (Qiagen Inc., Hilden, Germany), following the supplier’s instructions. The non-destructive protocol described in Vuataz et al.^38^, which enables post-extraction morphological study of specimens, was implemented where possible. For the largest specimens, total DNA was extracted from a leg. We then amplified a 658-bp fragment at the 5’ end of the mitochondrial cytochrome c oxidase subunit I gene, corresponding to the standard animal barcode region (CO1), using the HCO2198 and LCO1490 primers^39^. Polymerase Chain Reaction (PCR) was conducted in a volume of 25 to 33 μl, consisting of 5 to 9 μl (unknown concentration) of template DNA, 1.3 to 1.65 μl (10 μM) of each primer, 0.20 to 0.26 μl (25 mM) of dNTP solution (Promega), 5.0 to 6.6 μl of 5 to 10X buffer (Promega) containing 7.5 mM of MgCl_2_, 2.5 to 3.3 μl (25 mM) of MgCl_2_, 1.0 to 1.5 U of Taq polymerase (Promega), and 8.46 to 14.34 μl of sterile ddH_2_O. Optimized PCR conditions included initial denaturation at 95 °C for 5 min, 38 to 40 cycles of denaturation at 95 °C for 30 to 40 s, annealing at 50 °C for 30 to 40 s, and extension at 72 °C for 40 s, with final extension at 72 °C for 7 min. All PCR products were visualized after agarose gel electrophoresis to confirm amplicon size and detect possible contamination using negative controls. For a single specimen (GBIFCH00194305; *Perlodes dispar*; Table S2), a double band on the gel was obtained after migration of PCR products. Consequently, the band corresponding to the size of the barcode region was cut and purified from the gel before sequencing. Purification and automated sequencing were carried out in Microsynth (Balgach, Switzerland). Forward and reverse sequencing reads were assembled, quality-checked and edited in CodonCode Aligner 10.0.2 (Codon-Code Corporation, Dedham, MA).

**Figure 1:**
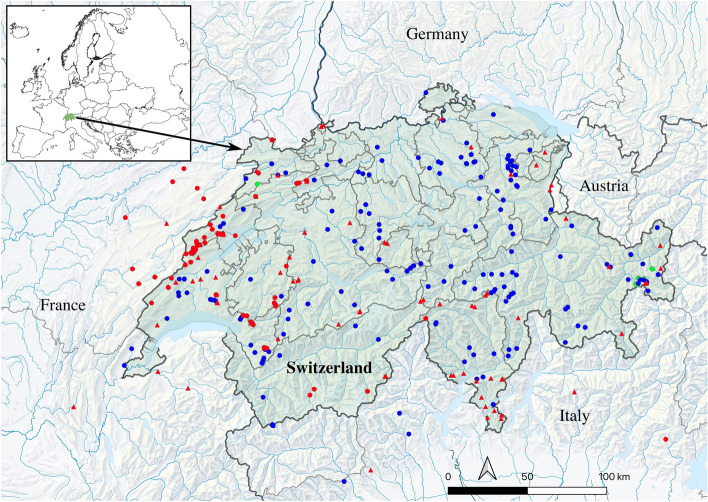
Sampling of Plecoptera in Switzerland (highlighted in green) and neighboring European countries. Red symbols indicate sampling sites of specimens with a newly published barcode sequence, with red circles corresponding to sites from AR^36^ and red triangles representing new sites. Blue circles indicate sampling sites from Gattolliat et al.^24^, while green circles correspond to sampling sites from GenBank. Seven localities in France and two in Italy fall outside the map's range. Land cover: Federal Office of Topography swisstopo.

We finally downloaded and added to our dataset all barcode sequences from stonefly specimens sampled in Switzerland available in GenBank as of August 2022. For this, we downloaded the sequences matching the ncbi query “plecoptera AND (co1 OR cox1 OR coi OR cytochrome oxidase) AND Switzerland” and deleted manually one sequence that was not sampled in Switzerland. We also checked for additional sequences in BOLD by downloading the seven TSV files (option “Combined: TSV”) matching queries corresponding to the seven families occurring in Switzerland (i.e. “Capniidae”, “Taeniopterygidae”, “Leuctridae”, “Nemouridae”, “Perlodidae”, “Perlidae”, “Chloroperlidae”), but no additional sequences could be obtained. We decided against including GenBank/BOLD sequences from species present in Switzerland but collected in adjacent countries. Morphological reexamination of the specimens from multiple studies would have been challenging or impossible, compromising data quality. Furthermore, including these sequences would have diluted the Switzerland-specific information, as there are already more than 1800 stonefly sequences from adjacent countries in GenBank, resulting in a fourfold increase in the dataset. Our final dataset includes 391 imagos (48% males; 51% females; 1% unknown) and 182 unsexed larvae. These specimens were collected by 26 contributors from May 1985 to September 2021 across 320 sites in Switzerland, 21 in France, and 8 in Italy, covering altitudes from 248 to 2450 m (Table S2). Our sampling map (Fig. 1) was created in QGIS 3.34^40^ and edited in Inkscape 1.3.2^41^.

### Genetic distances and gene tree reconstruction

All sequences were imported in Jalview 2.11.2.4^42^, aligned using MAFFT^43^ with default settings as implemented in Jalview, trimmed to the targeted 658 bp fragment and checked for stop codon. Unique haplotypes were obtained using Collapsetypes 4.6^44^.

Pairwise distances from CO1 sequences were calculated using the dist.dna function of the ape 5.7-1 package^45^ for R 4.2.3^46^, under the raw model and the pairwise.deletion option, corresponding to uncorrected p-distances (see Srivathsan and Meier^47^) with missing data removed in a pairwise way. Minimum, maximum and mean CO1 distances within and between CO1 putative species were calculated using the ddply function of the plyr 1.8.8 package^48^ for R. For testing the existence of barcoding gaps, which occur when the maximum distance within a species is smaller than the distance to the nearest species (i.e., nearest neighbor, NN hereafter; see Meier et al.^49^), maximum distances within CO1 putative species and distances to the NN were compared.

To improve both computational efficiency and the clarity of the subsequent CO1 gene trees, we divided our dataset into four subsets: Capniidae + Taeniopterygidae; Leuctridae; Nemouridae; Perlodidae + Chloroperlidae + Perlidae). Prior to gene tree reconstruction, the best evolutionary model was selected for each subset (HKY + Γ + I for Leuctridae and Perlodidae + Chloroperlidae + Perlidae; GTR + Γ + I for Capniidae + Taeniopterygidae and Nemouridae) following the second-order Akaike information criterion (AICc^50^) implemented in JModelTest 2.1.10^51^ with three substitution schemes, six gamma categories and all other parameters set to default. Bayesian inference as implemented in BEAST 1.10.4.^52^ was then used on the CIPRES Science Gateway 3.3^53^. For each analysis, the best model of evolution was implemented with 2 partitions (codon positions 1 + 2, 3) in BEAUTi 1.10.4^52^, with a relaxed clock model (uncorrelated with a lognormal distribution), a coalescent (constant size) tree model prior, a UPGMA starting tree, and two independent MCMC chains run for 50 million generations (sampled every 1000 generations). All other parameters were set to default. Run convergence was visually verified in Tracer 1.7.2^54^ and the independent log and tree files were combined using LogCombiner 1.10.4^52^ after discarding 10% of the trees as burn-in, whereby all parameters reached ESS values  > 200. The maximum clade credibility tree of each subset, obtained in TreeAnnotator 1.10.4^52^ with all options set to default, was visualized and edited in iTOL 6.5.7^55^.

### Morphological identification and species delimitation

For CO1 putative species delimitation, we employed two distinct approaches: one based on our prior morphological knowledge of species (CO1 clusters hereafter), and another utilizing computer-based statistical methods without preassigned species information, referred to as molecular operational taxonomic units (MOTUs) hereafter.

In the first approach, we initially assigned each barcoded specimen to a species or subspecies listed in Roesti^30^ based on its morphological characteristics. This was done using identification keys or characters provided in Aubert^56^, Ravizza and Vinçon^57^, Zwick^58^, Lubini et al.^59^, Reding^60^, and Roesti^30^. We employed morphology-based identifications as a reference to delimit and name CO1 clusters within the Bayesian gene trees, aiming to maximize congruence between morphology and the observed CO1 clades. An exact match (a single species name within a CO1 clade) was considered congruent, while cases of lumping (multiple species names within a clade), splitting (a single species name within multiple clades), or both, were considered incongruent. When incongruence was detected, we carefully blind-reexamined the specimens and made necessary corrections to the morphological identifications according to the latest taxonomical developments. The few specimens that posed morphological ambiguity, primarily larvae or females, were excluded from the congruence assessment.

In the second approach, we employed two contrasting single-locus species delimitation methods on our CO1 subsets: the distance-based ASAP (Assemble Species by Automatic Partitioning^61^) and the tree-based mPTP (multi-rate Poisson Tree Processes^62^) approaches. The ASAP method, which is an improvement of the widely used ABGD (Automatic Barcode Gap Discovery^63^) approach, has the advantage of providing a score that designates the most likely number of MOTUs. ASAP was separately applied to our four CO1 subsets using the ASAP webserver available at https://bioinfo.mnhn.fr/abi/public/asap/asapweb.html, computing the genetic distances under the default substitution model (JC69^64^). The best partition for species delimitation was selected based on the lowest asap-score within the genetic distance range of 0.005 to 0.05. The mPTP approach, which is a multi-rate extension of the PTP (Poisson Tree Processes^65^), exploits intra and interspecies phylogenetic differences, using the number of substitutions from a phylogenetic tree. We separately applied mPTP to our CO1 subsets using the web service available at https://mptp.h-its.org, using the BEAST CO1 gene trees as input (see above).

## Results

A total of 573 CO1 sequences (369 haplotypes) were included in this study: 253 (44.2% of the total) from Gattolliat et al.^24^, 151 (26.4%) newly sequenced here, 127 (22.2%) from AR (unpublished sequences^36^) and 42 (7.3%) from GenBank (one from Reding et al.^66^; one from Vinçon et al.^29^; 40 from Blattner et al.^67^). These were morphologically attributed to seven families, 20 genera and 119 species, fully covering the extant (i.e., not considered as locally extinct or doubtful) species reported from Switzerland according to Roesti^30^. While most of the sequences (518; 90.4% of the dataset) were from specimens sampled in Switzerland, 33 (5.8%) were from specimens sampled in France and 22 (3.8%) in Italy (Fig. 1; Table S2). Leuctridae was the most sequence-rich family with 202 sequences (35.3% of total; 125 haplotypes), followed by Nemouridae with 162 sequences (28.3%; 113 haplotypes), Perlodidae with 96 sequences (16.8%; 55 haplotypes), Capniidae with 37 sequences (6.5%; 20 haplotypes), Taeniopterygidae with 31 sequences (5.4%; 22 haplotypes), Chloroperlidae with 23 sequences (4.0%; 14 haplotypes) and Perlidae with 22 sequences (3.8%; 20 haplotypes). Our CO1 data matrix, which was  > 96% complete (3.6% missing data), included 301 variable sites (46%) and 272 parsimony-informative sites (41%). No insertion, deletion or stop codon was observed within the dataset.

**Figure 2:**
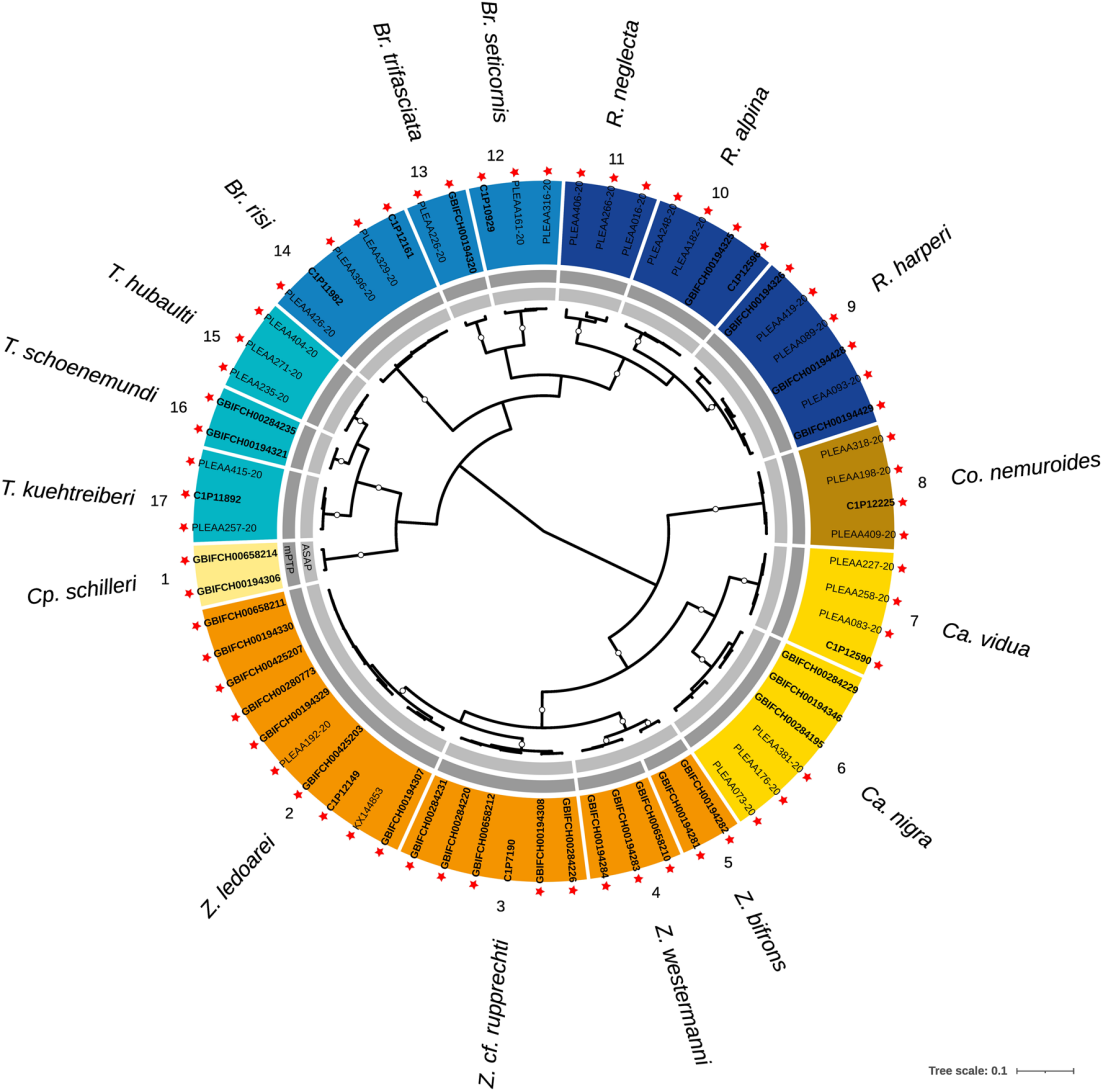
Bayesian (BEAST) maximum clade credibility CO1 tree of the Capniidae and the Taeniopterygidae families. Color ranges (outer ring) represent CO1 clusters delimited based on a priori morphological knowledge of species (color-coded by genus) and their associated numbers (1–8 for Capniidae; 9–17 for Taeniopterygidae) and names (outermost part). Black species names indicate cases of congruence between morphology and CO1 (a single species name within a CO1 clade). Abbreviations of genera: *Cp.*: *Capnopsis*; *Z.*: *Zwicknia*; *Ca*.: *Capnia*; *Co.*: *Capnioneura*; *R.*: *Rhabdiopteryx*; *Br.*: *Brachyptera*; *T.*: *Taeniopteryx*. The two inner grey rings indicate MOTUs delimited based on the two automated single-locus species delimitation methods (light grey: ASAP; dark grey: mPTP). Within the color ranges, tips are labelled according to the origin of the sequences (PLEAA codes: Gattolliat et al.^24^; C1P codes: Reding^36^; GBIF codes: newly produced barcodes for this study; other codes: GenBank), with bold labels for newly published sequences. Red stars indicate sequences from specimens sampled in Switzerland. Empty dots on branches represent posterior probabilities > 0.9

**Figure 3:**
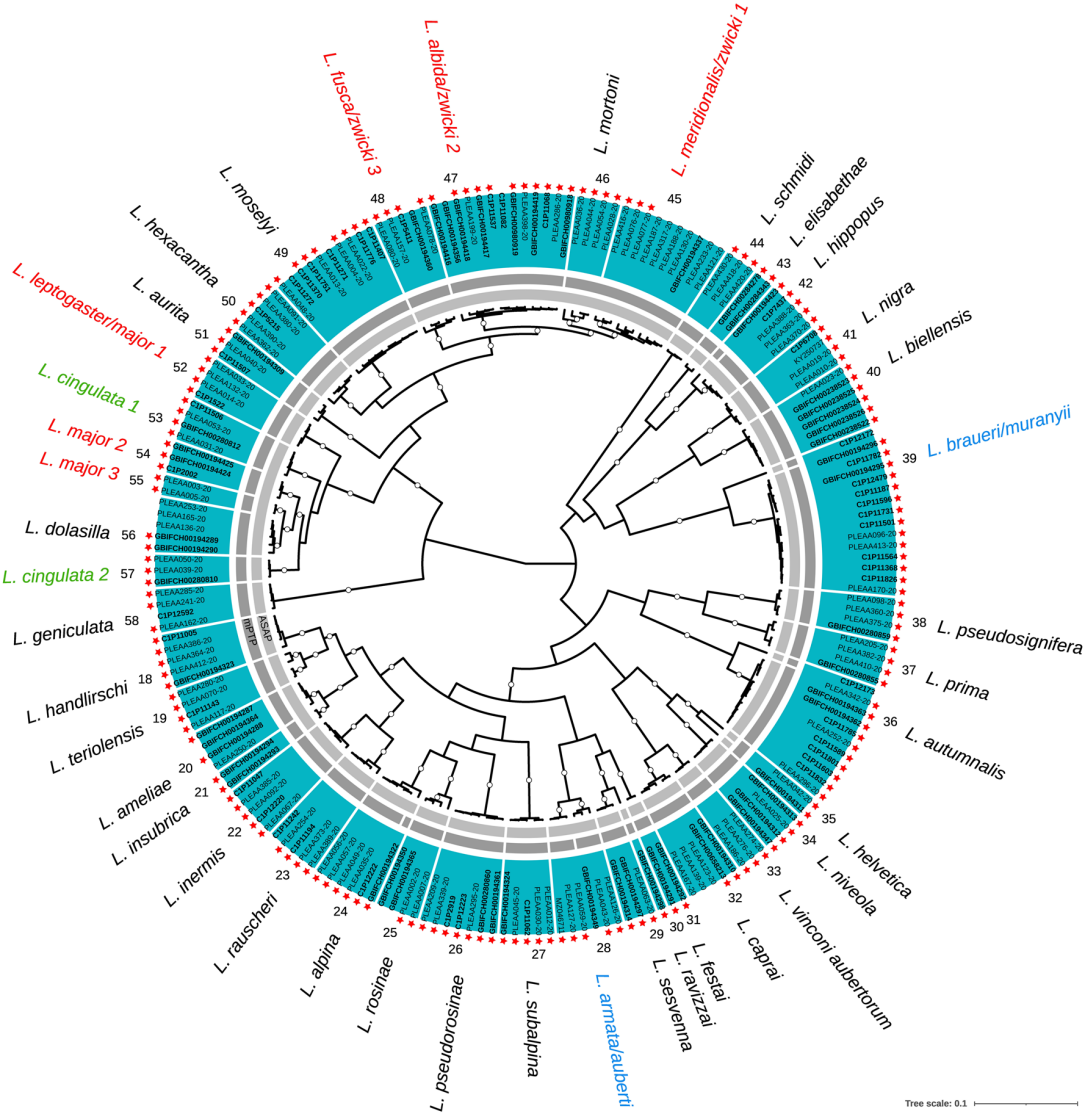
Bayesian (BEAST) maximum clade credibility CO1 tree of the Leuctridae family. Color ranges (outer ring) represent CO1 clusters delimited based on a priori morphological knowledge of species and their associated numbers and names (outermost part). Black species names indicate cases of congruence between morphology and CO1 (a single species name within a CO1 clade); blue shows cases of lumping (multiple species names within a clade); green indicates cases of splitting (a single species name within multiple clades); red shows both lumping and splitting. Abbreviation of the genus: *L.*: *Leuctra*. The two inner grey rings indicate MOTUs delimited based on the two automated single-locus species delimitation methods (light grey: ASAP; dark grey: mPTP). Within the color ranges, tips are labelled according to the origin of the sequences (PLEAA codes: Gattolliat et al.^24^; C1P codes: Reding^36^; GBIF codes: newly produced barcodes for this study; other codes: GenBank), with bold labels for newly published sequences. Red stars indicate sequences from specimens sampled in Switzerland. Empty dots on branches represent posterior probabilities > 0.9

**Figure 4:**
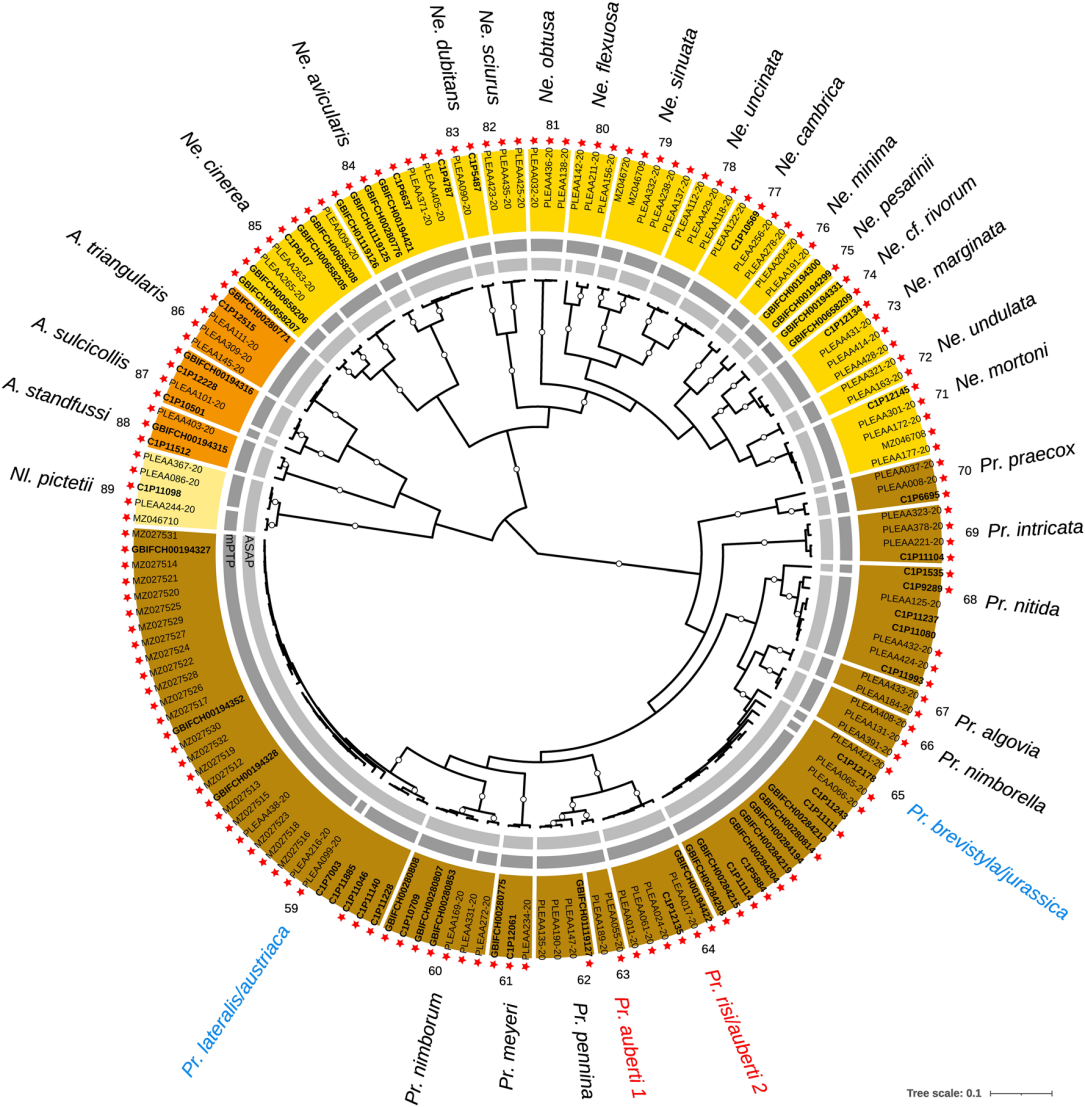
Bayesian (BEAST) maximum clade credibility CO1 tree of the Nemouridae family. Color ranges (outer ring) represent CO1 clusters delimited based on a priori morphological knowledge of species (color-coded by genus) and their associated numbers and names (outermost part). Black species names indicate cases of congruence between morphology and CO1 (a single species name within a CO1 clade); blue shows cases of lumping (multiple species names within a clade); red shows both lumping and splitting. Abbreviations of genera: *Pr.*: *Protonemura*; *Ne.*: *Nemoura*; *A*.: *Amphinemura*; *Nl.*: *Nemurella*. The two inner grey rings indicate MOTUs delimited based on the two automated single-locus species delimitation methods (light grey: ASAP; dark grey: mPTP). Within the color ranges, tips are labelled according to the origin of the sequences (PLEAA codes: Gattolliat et al.^24^; C1P codes: Reding^36^; GBIF codes: newly produced barcodes for this study; other codes: GenBank), with bold labels for newly published sequences. Red stars indicate sequences from specimens sampled in Switzerland. Empty dots on branches represent posterior probabilities > 0.9

**Figure 5:**
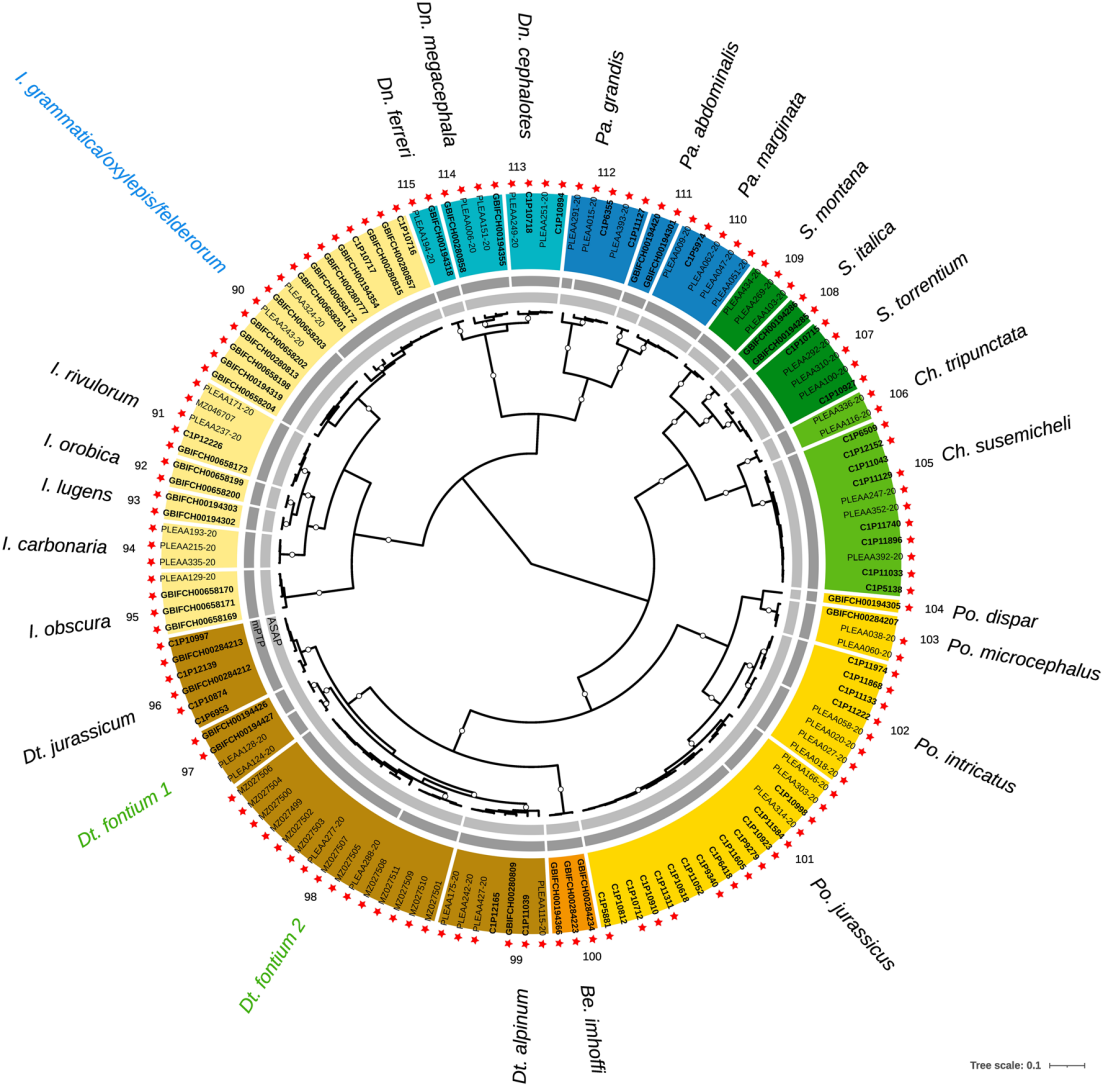
Bayesian (BEAST) maximum clade credibility CO1 tree of the Perlodidae, Chloroperlidae and Perlidae families. Color ranges (outer ring) represent CO1 clusters delimited based on a priori morphological knowledge of species (color-coded by genus) and their associated numbers (90–104 for Perlodidae; 105–109 for Chloroperlidae; 110–115 for Perlidae) and names (outermost part). Black species names indicate cases of congruence between morphology and CO1 (a single species name within a CO1 clade); blue shows cases of lumping (multiple species names within a clade); green indicates cases of splitting (a single species name within multiple clades). Abbreviations of genera: *I.*: *Isoperla*; *Dt.*: *Dictyogenus*; *Be*.: *Besdolus*; *Po.*: *Perlodes*; *Ch.*: *Chloroperla*; *S.*: *Siphonoperla*; *Pa.*: *Perla*; *Dn.*: *Dinocras*. The two inner grey rings indicate MOTUs delimited based on the two automated single-locus species delimitation methods (light grey: ASAP; dark grey: mPTP). Within the color ranges, tips are labelled according to the origin of the sequences (PLEAA codes: Gattolliat et al.^24^; C1P codes: Reding^36^; GBIF codes: newly produced barcodes for this study; other codes: GenBank), with bold labels for newly published sequences. Red stars indicate sequences from specimens sampled in Switzerland. Empty dots on branches represent posterior probabilities > 0.9

A total of 115 CO1 clusters (41 within Leuctridae, 31 within Nemouridae, 15 within Perlodidae, nine within Taeniopterygidae, eight within Capniidae, six within Perlidae and five within Chloroperlidae) were delimited and named in our gene trees according to a priori morphological species assignation (Figs. 2, 3, 4 and 5; Table S1 and S2). The number of sequences per CO1 cluster ranged from 1 to 32, with a mean of 5.0 sequences (i.e., specimens) and 3.2 haplotypes. The pairwise CO1 distances across all sequences ranged from 0 to 25.1%. The overall mean CO1 distance within CO1 clusters was 0.8%, whereas it was 1.6% (maximum 9.4%) for Nemouridae, 0.6% for Leuctridae, Perlodidae and Perlidae (maximum 8.1%, 4.9% and 2.8%, respectively), 0.4% for Taeniopterygidae and Chloroperlidae (maximum 1.9% and 1.6%, respectively) and 0.2% (maximum 1.8%) for Capniidae (Table S1). The mean distances within CO1 clusters were > 5% for two CO1 clusters (35 and 70). The overall mean CO1 distance between CO1 clusters was 18.6% (mean range 1.8–24.8%), whereas it was 20.9% (5.2–24.8%) for Perlidae, 19.5% (2.6–24.3%) for Perlodidae, 19.0% (8.7–22.9%) for Chloroperlidae, 18.9% (6.5–23.7%) for Taeniopterygidae, 18.7% (3.9–24.3%) for Nemouridae, 18.6% (2.1–22.8%) for Capniidae and 17.8% (1.8–24.8%) for Leuctridae. For 112 out of 115 CO1 clusters (97.4%), a DNA barcoding gap was observed. For CO1 clusters 46, 68 and 98 however, the maximum distance within CO1 clusters was greater than the distance to the NN, indicating an overlap between intra- and inter-CO1 cluster distance distributions (Table S1).

Among the 115 CO1 cluster, 98 (85.2%) corresponded to a single species (congruence between morphology and CO1). The 17 cases of incongruence resulted from lumping multiple species into a single CO1 cluster (5 cases; 4.3%), splitting a species into multiple CO1 clusters (4 cases; 3.5%), or both (8 cases; 7.0%). Most cases of incongruence were detected within Leuctridae (10), followed by Nemouridae (4) and Perlodidae (3). There was a perfect match between morphology and barcodes within Capniidae, Taeniopterygidae, Chloroperlidae and Perlidae (Figs. 2, 3, 4 and 5). A total of 19 CO1 clusters (16.5%), distributed within the seven families, were newly reported for Switzerland (i.e., excluding any already published sequence from specimens sampled in Switzerland). Among them, four CO1 clusters (3.5%) within the Leuctridae and Nemouridae families were never reported before (no species match in BOLD as of August 2022; sequence identity  < 95% in BLAST as of August 2022; Table S2).

A total of 112 MOTUs were delimited according to the ASAP method (Figs. 2, 3, 4 and 5; Table S2). Among them, 77 (68.8%) were congruent with morphology, 10 (8.9%) lumped, 19 (17.0%) split and 6 (5.4%) both lumped and split morphospecies. Incongruence was mainly detected within Nemouridae (18) and Leuctridae (12), followed by Perlodidae (3), Capniidae (1) and Perlidae (1). There was a perfect match between morphology and the ASAP method within Taeniopterygidae and Chloroperlidae. The mPTP method resulted in 137 MOTUs, with 82 (59.9%) cases of congruence with morphology, 0 lumped, 35 (25.5%) split and 20 (14.6%) both lumped and split morphospecies. Incongruence was mainly detected within Nemouridae (25) and Leuctridae (18), followed by Perlodidae (10) and Perlidae (2). There was a perfect match between morphology and the mPTP method within Capniidae, Taeniopterygidae and Chloroperlidae.

## Discussion

### The importance of morphology in single-locus DNA barcoding

The comparison between CO1 clusters and morphology indicated that our CO1 barcodes allow an unambiguous association with 85% of stonefly species in Switzerland. Similar results have been obtained for stoneflies in Croatia^68^ and Germany^4^, with congruence rates of 84% and 88% between CO1 and species, respectively, and for other insects as well (e.g., 85% and 88% congruence in Odonata of Italy^69^ and Central Europe^70^, respectively; 89% congruence in Diptera of Germany^71^). Moreover, a local barcoding gap was observed in over 97% of the CO1 clusters, suggesting that a simple distance-based approach is sufficient for accurately associating most barcodes with species. In the age of genomics, traditional CO1-based barcoding remains thus a valuable tool for identifying stonefly species in Switzerland. However, achieving accurate results requires careful use and interpretation of the reference database due to the presence of ambiguity in 15% of the associations between CO1 clusters and morphology. In some cases, up to three species may be associated with a single CO1 cluster, and vice versa (Figs. 3 and 5; Table S2).

Remarkably, when utilizing a-priori morphological knowledge as a reference, our CO1 putative species exhibited much higher congruence with species (85%) compared to using automated species delimitation methods (69% congruence with ASAP and 60% with mPTP). Specifically, automated clustering methods showed significant discrepancies in MOTUs delimitation for Leuctridae, Nemouridae, and Perlodidae (Figs. 3, 4 and 5). Despite the increasing availability of single-locus species delimitation methods, the consensus from the plethora of studies comparing these algorithms is that they consistently yield different results^72,73^, suggesting simple variations around a single, potentially biased signal^74^. While comparing multiple methods helps assess signal consistency and potentially establish a reasonable consensus, challenges persist due to the complications associated with using a single mitochondrial marker (see below). Incorporating reliable morphological knowledge in barcoding studies undeniably helps overcome challenges posed by fluctuating results from automated algorithms, allowing for clear differentiation between cases of incongruence and perfect matches^75^. Future investigations involving multiple and complementary data (integrative taxonomy) can thus become more effective by concentrating on the highlighted incongruence cases.

### Limitations and progress in DNA barcoding

While the association of a CO1-based approach and solid morphological knowledge optimizes conventional barcoding, the limitations of single-locus methods persist across many taxonomic groups. These drawbacks encompass various biological mechanisms that affect congruence between gene trees and species trees, such as the retention of ancestral polymorphism (incomplete lineage sorting) and gene flow across species boundaries, including the transfer of mitogenomes between species through hybridization (see Funk and Omland^76^ for a review of causes and consequences of gene-tree/species-tree discordance). Endosymbiotic bacteria, such as *Wolbachia*, can amplify the effects of introgressions on the accuracy of DNA barcoding by manipulating host reproduction and potentially causing a decrease in intraspecific barcode variability^77,78^. Conversely, pseudogenes (numts), which are paralogous nonfunctional mitochondrial fragments integrated into the nuclear genome^79^, have the potential to artificially inflate haplotype richness and species diversity when co-amplified with universal primers^80^. Multilocus DNA barcoding^75,81–83^ offers a promising solution to these challenges, benefiting from ongoing advancements in next generation sequencing methods associated with reduced costs and improved accessibility. By particularly targeting multiple nuclear loci, these approaches have proven to be more successful than traditional methods in accurately delineating closely related species, while circumventing the problem of mitochondrial introgression^84^. Its broad acceptance and full deployment depend on the resolution of remaining challenges, including the need for universality, adequate variability and backward compatibility of targeted regions, improved accessibility to analytical routines, and competitive costs.

### A taxonomic exploration of incongruence

The 17 cases of incongruence identified offer valuable opportunities for further study. Some of these cases are already known or currently being investigated, while others are revealed here. In addition to the various mechanisms that can affect the congruence between CO1 putative species and morphology (see previous section), there are more straightforward explanations for either lumping or splitting species. One obvious explanation for cases of lumping is the presence of unrecognized species synonymy, especially when dealing with closely related species that pose challenges in morphological differentiation. Conversely, the simplest explanation for cases of splitting is the existence of unrecognized species diversity, which may involve new species for the country or region, or new to science. A second important explanation to be considered here is sampling artifacts, where oversplitting of species can occur when intermediate haplotypes are missing due to large unsampled geographic areas^85–87^. However, accurate morphological identifications should ensure correct species delimitation in such situations.

In the following discussion, we briefly examine each case, highlighting potential directions for future taxonomic studies. The cases are presented in ascending order of CO1 cluster numbers from Figs. 3, 4 and 5.

- CO1 cluster 28: lumping of *Leuctra armata* and *Leuctra auberti* (Leuctridae; Fig. 3)

*Leuctra auberti*, a species endemic to Italy^88^, is described from the Pennine Alps, and shares obvious morphological similarities with *L. armata* with which it was formerly confused^89^. The barcode of the single *L. auberti* specimen included in this study (GBIFCH00194349; Table S2), sampled in the Lombardian Alps at a location less than 30 km from Switzerland, was lumped with the *L. armata* barcodes from specimens sampled in Graubünden, Switzerland. Additional research is necessary to understand the factors behind this grouping and to investigate the potential future colonization of Switzerland by *L*. *auberti*.

- CO1 cluster 39: lumping of *Leuctra braueri* and *Leuctra muranyii* (Leuctridae; Fig. 3)

*Leuctra braueri* and *L. muranyii* are closely related species. While the former species is widespread in the Alps, the latter species, previously confused with *L*. *braueri*, is described mainly from the southeastern Alps and is found in Switzerland only in the far eastern region of the country^30^. Interestingly, the single *L*. *muranyii* barcode from Italy (C1P12172; Table S2) was recovered as sister to all the other *L*. *braueri* and *L*. *muranyii* barcodes (all from specimens collected in Switzerland), yet there was a significant genetic distance between them (p-distance: 7.5–8.1%). Further investigations are required to clarify the reasons for this grouping and to understand the implications of this genetic differentiation.

- CO1 clusters 45, 47, 48: both lumping and splitting of *Leuctra meridionalis*; *Leuctra albida*; *Leuctra fusca*; *Leuctra zwicki* (Leuctridae; Fig. 3)

The 11 barcodes attributed to *L*. *zwicki* were divided into three CO1 clusters: one including all sequences attributed to *L*. *meridionalis* (CO1 cluster 45), one with all sequences attributed to *L*. *albida* (CO1 cluster 47), and another with all sequences attributed to *L*. *fusca* (CO1 cluster 48). *Leuctra zwicki*, originally described from the Mercantour in France^90^, is a Prealpine species endemic to the southern part of the western Alps, with its northern-most range limit in the Swiss Jura Mountains. As part of the *L*. *fusca* subgroup, these four species share close morphological similarities. Gattolliat et al.^24^ reported the inability to distinguish *L*. *meridionalis* from French populations of *L*. *zwicki* using CO1 alone, suggesting hybrid introgression as a potential explanation, and suggested the role of the climatic oscillations of the Pleistocene in the recent speciation of closely related species pairs like *L*. *albida* (northern slope of the Alps) and *L*. *meridionalis* (southern slope). Our findings show that *L*. *zwicki* CO1 barcodes cannot be distinguished from *L*. *albida* and possibly *L*. *fusca*, although confirmation is needed for the latter case involving a single *L*. *zwicki* female imago (Table S2). Further research is needed to better understand the intricate evolutionary history and relationships between *L*. *zwicki* and the other members of the *L*. *fusca* subgroup.

- CO1 clusters 52, 54, 55: both lumping and splitting of *Leuctra leptogaster* and *Leuctra major* (Leuctridae; Fig. 3)

One female imago from Switzerland (Canton of Lucerne; code PLEAA132-20; Table S2) included in CO1 cluster 52 was morphologically identified as *L*. *major*, while all other specimens within the same cluster were identified as *L*. *leptogaster*. The other barcodes associated with *L*. *major* were split into two distinct CO1 clusters, 54 and 55. Interestingly, sequences in CO1 cluster 54 were from specimens collected in the Swiss Jura mountains and Prealps, while sequences in CO1 cluster 55 were from specimens sampled in (or very close to) the Swiss internal slope of the Alps. A comparison with sequences available in BOLD as of August 2022 revealed that four barcodes from *L*. *major* specimens collected in Vorarlberg, Austria (Code INTAP001-17–INTAP004-17) were highly similar (99.7% to 100%) to those in CO1 cluster 55. A BLAST search returned several sequences from specimens sampled in Croatia^68^, Germany^4^ and Italy^91^ with 98.0% to 98.9% similarity, suggesting more complex CO1 relationships within the *L. major* taxonomical concept that require further investigation. No good match was obtained in public repositories for the barcodes in CO1 cluster 54.

- CO1 clusters 53, 57: splitting of *Leutra cingulata* (Leuctridae; Fig. 3)

CO1 cluster 53 exclusively consists of specimens from the Swiss Jura Mountains, while CO1 cluster 57 includes specimens from the Swiss Plateau, Prealps, and internal slope of the Alps. The geographical structuring between these two clusters in Switzerland, their relatively high genetic distance (5.3%), and their separation in the gene tree all suggest the presence of two distinct species rather than a sampling artifact. However, despite a preliminary morphological examination, no obvious characteristics were found to distinguish them, and their occupation of the same larval ecological niche (hyporheic habitats in steep brook sections or springs) indicates they could be cryptic species. A BLAST search on our sequences in CO1 cluster 53 returned identical or nearly identical sequences from specimens identified as *L*. *cingulata*, collected in the western French Alps^92^. Interestingly, we also obtained a 98.7% identical sequence from a specimen sampled in the French Massif Central, identified as *Leuctra pseudocingulata*^92^. As *L*. *pseudocingulata* is reported exclusively from crystalline ranges and not expected in the Jura Mountains, we don't believe CO1 cluster 53 corresponds to this species. Similarly, for CO1 cluster 57, the same query returned five sequences (98.9% to 100% identical) identified as *L*. *cingulata*, but from specimens sampled in the eastern German Alps^4^. Additional research is necessary to address this case and explore the possibility of describing a new species.

- CO1 cluster 59: lumping of *Protonemura lateralis* and *Protonemura austriaca* (Nemouridae; Fig. 4)

*Protonemura lateralis* and *P*. *austriaca* are morphologically similar, with only subtle features allowing their differentiation^30^. While the former species is common and widespread throughout Switzerland, the latter species was reported in Switzerland only in 2018 by GV from a single locality in the far east of the country (Canton of Graubünden), representing its known western distribution limit. The far east of Switzerland could potentially be a hybrid zone where gene flow between both species still occurs. Further investigation is needed to better understand the relationship between the two species and their dynamics in this region.

- CO1 clusters 63, 64: both lumping and splitting of *Protonemura auberti* and *Protonemura risi* (Nemouridae; Fig. 4)

The only problematic specimen in CO1 cluster 64 is one female imago from Switzerland (Canton of St. Gallen; code PLEAA017-20; Table S2) morphologically identified (both pre- and post-DNA extraction) as *P*. *auberti*, while all other specimens in the cluster were identified as *P*. *risi*. Although both species are closely related and were confused in the past^93^, female imagos have unambiguous morphological differences (e.g., reliable features on subgenital plate and vaginal lobes), and we are confident that this incongruence is not due to misidentification. Further investigations will help clarify this intriguing case.

- CO1 cluster 65: lumping of *Protonemura brevistyla* and *P. jurassica* (Nemouridae; Fig. 4)

Recently described based on morphological, geographical, and ecological evidence, *P*. *jurassica* is an endemic species from the Jura Mountains that was previously confused with *P*. *brevistyla*^27^. Interestingly, the gene tree topology within CO1 cluster 65 supported all barcodes associated to *P. jurassica* as monophyletic, although without a strong support, while *P*. *brevistyla* barcodes were paraphyletic. In future investigations utilizing multilocus approaches, we anticipate that both species will be recovered as monophyletic.

- CO1 cluster 90: lumping of *Isoperla grammatica*; *Isoperla oxylepis*; *Isoperla felderorum* (Perlodidae; Fig. 5)

*Isoperla* is one of the most species-rich stonefly genera^94^. It is morphologically challenging, particularly the *I*. *grammatica* species group^95,96^. However, *I*. *grammatica* and *I*. *oxylepis* can be morphologically separated by their penial armature, which is narrow and elongated in the former (Despax^97^, plate III, Fig. 10), whereas it is wider and shorter in the latter (Despax^97^, plate III, Fig. 12). Moreover, the scales of the penial armature are mucronate in *Isoperla grammatica* (Despax^97^, plate III, Fig. 21), whereas these scales are narrow and acuminated in *I. oxylepis* (Despax^97^, plate III, Fig. 23). Both species can also be distinguished by their drumming signals. Recently described from Switzerland, *I*. *felderorum* was since then confused with *I. grammatica*, which is morphologically very similar^30^. Nevertheless, the two species can be differentiated based on their drumming signals.

- CO1 clusters 97, 98: splitting of *Dictyogenus fontium* (Perlodidae; Fig. 5)

We obtained two distinct clusters composed of *D*. *fontium* specimens, with a mean genetic distance of 2.8% between them. CO1 cluster 97 comprises specimens from the western Swiss Alps (Cantons of Valais and Berne) and Italy (Aosta Valley near Quincinetto), while CO1 cluster 98 consists of specimens from the Swiss Rhaetian (eastern) Alps (Canton of Graubünden). Advancements have recently occurred in the taxonomic revision of the genus *Dictyogenus*, with six new species described from the Austrian, Italian, Slovenian, and Swiss Alps^98^. Considering the geographical origin of the specimens, CO1 cluster 97 is likely to include both *D*. *fontium* and the new species *D*. *padanum*, while CO1 cluster 98 would correspond to the new species *D*. *nadigi*, except for PLEAA288-20 from the Canton of Graubünden. To clarify the utility of CO1 in delimiting the six new *Dictyogenus* species and to resolve the complex relationships among these closely related species, further research encompassing the entire range of the genus is necessary.

### Potential hidden diversity

High CO1 distances within CO1 clusters could suggest the existence of hidden diversity. Therefore, we recommend a more thorough examination of *Leuctra helvetica*, and *Protonemura praecox* in the future, given the substantial mean distances within the associated clusters (> 5%; Table S1). We also advise further research on *Leuctra autumnalis*, *Protonemura nitida*, *Nemoura sinuata*, *Nemoura avicularis*, *Nemoura cinerea*, and *Amphinemura sulcicollis*, as the two automated species delimitation methods agreed on distinguishing separate entities for the associated clusters. Additionally, we labeled two CO1 clusters with uncertainty using “cf.” notation (*Zwicknia* cf. *rupprechti* and *Nemoura* cf. *rivorum*). These might correspond to species new to science and are briefly discussed below.

- CO1 cluster 3: *Zwicknia* cf. *rupprechti* (Capniidae; Fig. 2)

*Zwicknia rupprechti* has been described from Croatia and Hungary^99^. Boumans and Murányi^100^ reported a new, distinct CO1 clade comprising sequences from specimens sampled in France and Germany (referred to as “northwestern populations”) provisionally associated to *Z. rupprechti* based on morphology and drumming behavior. The species, associated to crystalline massifs relicts of the Hercynian orogeny^101^, is not expected in Switzerland^31^, except in the distant foothills of the Vosges Mountains or the Black Forest marginally extending into Switzerland. A BLAST search as of August 2022 on our sequences in CO1 cluster 3, which is exclusively composed of specimens from the Swiss Jura Mountains (Table S2), returned seven sequences with up to 99.7% similarity. These are from the putative “northwestern” populations of *Z. rupprechti* published in Boumans and Murányi^100^. More investigation, including the analysis of drumming signals, is necessary to clarify whether the Swiss Jura population corresponds to *Z. rupprechti*, or to a new, potentially cryptic species.

- CO1 cluster 74: *Nemoura* cf. *rivorum* (Nemouridae; Fig. 4)

This cluster corresponds to the species *Nemoura* sp. in Roesti^30^ and includes sequences from two specimens sampled in the easternmost part of Switzerland (Canton of Graubünden; Table S2). A preliminary morphological examination of those specimens indicated affinities with *Nemoura rivorum* described from the northern Apennines in Italy^102^. The query “Nemoura rivorum” in BOLD as of August 2022 returned two sequences from two localities in the Vorarlberg (Germany), ca. 80 km from our sampling site. These sequences exhibited a perfect match (100% similarity) to ours, supporting conspecificity of specimens. A BLAST search on our sequences returned one 100% and one 99.9% identical sequence, both from specimens identified as *Nemoura marginata* and published in a barcoding study on aquatic insects of Germany^4^. The authors report a high intraspecific genetic divergence (9.51%) for their *N. marginata* CO1 cluster, supporting the occurrence of multiple, undetected species within this clade. Similarly, the sequences associated to *N. marginata* in a recent barcoding study of Croatian stoneflies^68^ are from 96.0% to 99.1% identical to barcodes in CO1 cluster 74. Interestingly, Hlebec et al.^68^ defined a CO1 cluster composed of sequences they associated to both *N.* cf*. rivorum* and *Nemoura flexuosa*, which are closely related to barcodes in CO1 cluster 80 (“*Ne. flexuosa*”; Fig. 4) with up to 96.8% of identity. Hlebec et al.^68^ highlighted the high level of intraspecific morphological variability within *N.* cf. *rivorum* and *N. flexuosa*, and more generally the need for a taxonomical revision of the *N. flexuosa*-*marginata* complex. We agree that further investigation, including the barcoding of specimens from Italian populations, is needed to fully clarify the species delineation within this complex, and to confidently attribute CO1 cluster 74 to a described or a new species.

### Supplementary Information

Supplementary Information 1.

Supplementary Information 2.

## Data Availability

All CO1 barcodes newly published in this study are deposited in the NCBI GenBank database (https://www.ncbi.nlm.nih.gov/genbank/) under the accession numbers OR733730–OR734007. Information on specimens and associated barcodes, including identifications and GBIF codes, are provided in Table S2. The investigated specimens are housed in the Department of zoology, State Museum of natural sciences (Muséum cantonal des sciences naturelles) in Lausanne, Switzerland; they are readily accessible for on-site examination upon request.
